# Book Reviews

**Published:** 1903-06

**Authors:** 


THE AMERICAN YEAR BOOK OF MEDICINE AND SURGERY FOR 1903. A yearly Digest of Scientific Progress and Authoritative Opinions in all branches of Medicine and Surgery, drawn from journals, monographs and textbooks of the leading American and foreign authors and investigators. Arranged, with critical editorial comments, by eminent American specialists, under the editorial charge of GEORGE M. GOULD, A.M., M.D. In two volumes—Volume I. including General Medicine. Octavo, 700 pages, fully illustrated. Volume II., General Surgery. Octavo, 670 pages, fully illustrated. Philadelphia, New York and London: W. B. Saunders & Co. 1903. [Per volume: cloth, $3.00 net; half-morocco, $3.75 net.]

This work continues to be unique, in the way of condensations of annual medical literature. The excerpts are carefully made by men who are familiar with the publications of the year, and have a practical knowledge of what is most needed as well as what is best. It is not easy to make selections from among such a mass of material as publishers put out every year, but from our examination of the volumes we are of the opinion that rare discretion has been exhibited in the preparation of this year book. The chaff has been winnowed most thoroughly, leaving only the pure, clean grain behind.

The volume on medicine is slightly larger than that upon surgery, but both are suited in size for the greatest convenience of research. Illustrations have been used both as plate inserts and text cuts, with the effect of giving tone and zest to the pages of reading matter. All in all, W. B. Saunders & Company deserve commendation jointly with the editor, for bringing out one of the best works of its kind published in this country, if not in the world.

---

Clinical Treatises on the Pathology and Therapy of the Disorders of Metabolism and Nutrition. By Prof. Dr. Carl von Noorden, Senior Physician to the City Hospital in Frankfurt, a. M. Authorised American edition, translated under the direction of Boardman Reed, M.D., Professor of the Diseases of the Gastrointestinal Tract, Hygiene, and Climatology, Department of Medicine, Temple College, Philadelphia. Part I. Obesity; the Indications for Reduction Cures. Duodecimo, pp. 59. New York: E. B. Treat & Co. 1903. [Price, 50 cents.]

This is a handsomely executed monograph on a subject of interest to overweight people, and gives physicians useful hints concerning reduction measures that may be adopted without detriment to the individual. It will well repay careful reading by all physicians interested in the subject of obesity from a therapeutic viewpoint.

---

Blakiston's Quiz Compends. A Compend of Diseases of Children. Especially adapted for the use of Medical Students. By Marcus P. Hatfield, A.M., M.D., Emeritus Professor of Diseases of Children, Northwestern University Medical School; Physician to Wesley Hospital, Home for Crippled Children, Chicago Orphan Asylum, Chicago. Duodecimo, pp. 241. Third edition, thoroughly revised, with colored plate. Philadelphia: P. Blakiston's Son & Co. 1903. [Price, 80 cents.]

The object of this compend is to supply the undergraduate with an aid to his finals, and the post-graduate with similar aid in state examinations for license to practise. It is based upon a translation from the German of Kormans "Compendium der Kinderkrankheiten," made by the author and a fellow student while attending the University of Berlin several years ago.

It is an excellent résumé of pediatrics and meets the purpose in view with admirable effect and force. We challenge the propriety of such a sentence as this: "might substitute bismuth for magnesia, if much vomiting." (P. 198). Conciseness at the expense of grammar is undesirable. Some of the prescriptions are given in the metric system and others in apothecaries' weights and measures. Until the official establishment of the former it would be well to give formulæ in both.

A TEXT-BOOK OF PRACTICAL MEDICINE. For the Use of Students and Practitioners. By WILLIAM GILMAN THOMPSON, M. D., Professor of Medicine in Cornell University Medical College, Physician to the Presbyterian Hospital, Bellevue Hospital, New York. New (second) edition, thoroughly revised. In one 8vo volume of 1,104 pages, with 62 illustrations. Philadelphia and New York: Lea Brothers & Co. 1903. [Cloth, $5.00; leather, $6.00; half morocco, $6.50 net.]

When Professor Thompson's first edition appeared, now nearly three years ago, it at once attracted attention as a text-book on internal medicine, assuming promptly a place in the front rank of such literature; this, too, in spite of the fact that the market seemed pretty nearly overcrowded. Now, after sufficient time has elapsed not only to exhaust the first edition, but to permit the book to gravitate to its appropriate level, we still find it a favorite among teachers, students and practitioners. Nor is it difficult to understand or appreciate this fact when we again give the treatise a careful examination.

It is proper to remark that the second edition exhibits abundant evidence of careful revision, which is shown not only by many minor changes throughout the text, but by the more material alterations pertaining to the greater subjects of dysentery, yellow fever, malaria, and the like. In view of the great progress recently made in the study of these and other infectious diseases, especially as relates to cause and prevention, the material herein contributed by Professor Thompson will prove of great interest, and should command patient, thoughtful attention.

In noticing the first edition we called attention to the skill with which the author handled diseases of the blood and vascular system. In this edition there is evidence of careful revision of this important subject, and we note also that much new material has been added to the department of diseases of the digestive system. These and other points indicate a progressiveness on the part of the author which is in keeping with the spirit of the age, and which must influence the person who contemplates the purchase of a work on internal medicine. It is important in these busy days to obtain the very best information on a given subject, and to this book one will not appeal in vain for a fund of information in the field which it covers so well.

---

PROGRESSIVE MEDICINE. Fifth annual series. Volume I., March, 1903 A Quarterly Digest of Advances, Discoveries and Improvements in the Medical and Surgical Sciences. Edited by HOBART AMORY HARE, M. D., Professor of Therapeutics and Materia Medica in the Jefferson Medical College of Philadelphia. Octavo, 450 pages, illustrated. Philadelphia and New York: Lea Brothers & Co. [Per volume, $2.50, by express prepaid; per annum, in four cloth-bound volumes, $10.00.]

In this volume we find considered, first, surgery of the head, neck, and chest, by Charles H. Frazier; second, infectious diseases, including acute rheumatism, croupous pneumonia, and influenza, by James B. Herrick; third, diseases of children, by Floyd M. Crandall; fourth, pathology, by Ludvig Hektoen; fifth, laryngology and rhinology, by A. Logan Turner; and, sixth, otology, by Robert L. Randolph.

Like the preceding volumes this one is not simply a presentation of the several subjects in epitomé, but each is a carefully prepared dissertation, grouping and welding the best literature gleaned from the most trustworthy sources, and molded into a homogeneous compact form by experienced men, who add their own knowledge of each topic; making thereby a well digested encyclopedic article in each instance.

Like its predecessors, too, this book is admirably printed in large, clear type, upon tinted, well-finished paper, and is strongly bound, thus making an exceedingly agreeable volume to handle—not an insignificant item in the search for material on any subject within its covers.

